# Cilostazol improves endothelial function in acute cerebral ischemia patients: a double-blind placebo controlled trial with flow-mediated dilation technique

**DOI:** 10.1186/s12883-017-0950-y

**Published:** 2017-08-29

**Authors:** Seong-Joon Lee, Jin Soo Lee, Mun Hee Choi, Sung Eun Lee, Dong Hoon Shin, Ji Man Hong

**Affiliations:** 1 Department of Neurology, Ajou University School of Medicine, Suwon, Republic of Korea; 2 Department of Neurology, Gachon University Gil Hospital, Incheon, Republic of Korea; 3 Department of Neurology, Ajou University School of Medicine, 164, World cup-ro, Yeongtong-gu, Suwon-si, Gyeonggi-do 16499 South Korea

**Keywords:** Endothelium, Cilostazol, Cerebral infarction, Transient ischemic attack, Arginine

## Abstract

**Background:**

In order to evaluate the impact of cilostazol on endothelial function, we compared the changes of flow-mediated dilation (FMD) between aspirin and cilostazol groups in patients with acute cerebral ischemia.

**Methods:**

Patients presenting with acute cerebral ischemic events were randomly assigned into aspirin (*n* = 40) or cilostazol (*n* = 40) group in a double-blinded manner. FMD was measured at baseline (T0) and 90 days (T1). We measured L-arginine at baseline (a precursor of biologically active nitric oxides). Serious and non-serious adverse events were described.

**Results:**

Despite no difference in the baseline FMD values (*p = 0.363*), there was a significant increase of FMD values in cilostazol group (7.9 ± 2.4 to 8.9 ± 2.3%, *p = 0.001*) and not in aspirin group (8.5 ± 2.6 to 9.3 ± 2.8%, *p = 0.108*). In the multiple regression analysis performed in cilostazol group, serum L–arginine levels were inversely correlated with FMD at T1 (ß = −0.050, SE: 0.012, *p < 0.001*) with age, total cholesterol levels, and C-reactive protein as confounders. While T0 FMD values in both aspirin and cilostazol groups did not show any correlation with serum L-arginine levels, the correlation is restored in the cilostazol group at T1 (*r* = 0.467, *p = 0.007*), while such is not shown in the aspirin group. There was no difference of serious adverse events between the two groups (*p = 0.235*). Adverse events were more common in the cilostazol group (35/40 vs. 25/40, *p = 0.010*), due to frequent headaches (14/40 vs. 3/30, *p = 0.003*) which was well tolerated.

**Conclusion:**

Cilostazol improved endothelial function in acute cerebral ischemia patients. It also restored an inverse correlation between 3-month FMD and baseline L-arginine levels.

**Trial registration:**

NCT03116269, 04/12/2017, retrospectively registered.

**Electronic supplementary material:**

The online version of this article (10.1186/s12883-017-0950-y) contains supplementary material, which is available to authorized users.

## Background

Cilostazol is a selective phosphodiesterase (PDE) 3 inhibitor with therapeutic focus on cyclic adenosine monophosphate (cAMP) [1]. It inhibits platelet aggregation and plays a role as a direct arterial vasodilator [2, 3]. It prevents the occurrence of restenosis after coronary angioplasty and stenting and reduced growth of carotid intima-media thickness in diabetic patients [4, 5]. In cerebrovascular disease, the progression rate of symptomatic intracranial artery stenosis was significantly lower in the cilostazol aspirin combination group than in the aspirin monotherapy group [6]. These findings suggest antiatherogenic effects of cilostazol, previously known to be through actions on PDE3 in vascular smooth muscle cells [7], lipoprotein metabolism [8], and prevention of endothelial apoptosis [9].

In the development of atherosclerosis, endothelial dysfunction is a key step in its development and can be used as a prognostic marker in predicting future stroke and heart attacks [10, 11]. Endothelial function can be noninvasively assessed by the ultrasound measurement of brachial artery flow-mediated dilation (FMD), which represents the endothelium-dependent relaxation of an artery in response to reactive hyperemia [12]. FMD is comprised of two cellular mechanisms, endothelial nitric oxide (NO) synthesis from its precursor L-arginine, by mechanical shear-stress of vessel wall [13], and concurrent vascular smooth muscle cell relaxation through actions of NO. Accordingly, improvement of FMD can be a biomarker for protection against atherogenesis.

Therefore, we hypothesized that in acute cerebral ischemia patients, antiatherogenic effects of cilostazol can be represented by improvements in flow-mediated vasodilation, representing its positive effects in endothelial function. To achieve this goal, in acute stroke patients randomized to aspirin control group versus cilostazol group in a double-blinded manner, the value of FMD at admission, 3 months, and its improvement was compared, along with its association with baseline L-arginine levels as well as other known vascular risk factors.

## Methods

### Study design and participants

This investigator-initiated, randomized, double–blind trial was prospectively conducted with two-arm parallel treatment groups and a single dose scheme: 100 mg aspirin daily and cilostazol placebo twice daily versus aspirin placebo daily and 100 mg cilostazol twice daily. The dosage of each drug was planned according to previous prospective studies [1, 6, 14]. Randomization was performed by a computer generation block card randomization procedure. This study was conducted from March 2012 to October 2014. The institutional review board of Ajou University Hospital approved the protocol and all participants provided written informed consent.

Eligible patients were over 30 years of age and had an (1) acute ischemic stroke confirmed by diffusion weighted imaging, or a (2) transient ischemic attack (TIA) within 7 days. Patients were excluded if (1) there is intracranial hemorrhage on imaging study, (2) patients is previously taking antiplatelets, vitamin K antagonists, factor Xa antagonists, or chronic treatment with systemic steroidal and non-steroidal anti-inflammatory drugs, (3) patients who received fibrinolytics within the previous 48 h, (4) cognitive impairment interfering with the possibility of obtaining informed consent, (5) pregnancy, (6) participation in another pharmacological study, (7) peptic ulcer disease or hematological abnormality, (8) initial modified Barthel index <30 points, and (9) liver function tests exceeding a 2-fold upper range value. A total of 80 eligible patients were prospectively recruited. All included patients underwent diagnostic studies including routine blood tests and cardiologic work-ups. The primary outcome was differences in endothelial function in the two groups measured by means of FMD on admission and at 3 months. According to previous studies, the adverse effects in two groups were investigated. A flowchart for the study population is shown in Additional file 1: Figure S1.

### Flow-mediated dilation (FMD) protocol

Endothelial function was evaluated by measurement of FMD of the brachial artery in response to hyperemia according to methods previously described [15]. The subjects were instructed to fast after dinner on the day before assessment and to refrain from smoking, alcohol, or caffeine 6 h before the start of evaluation. They were fully rested before the FMD measurements. Briefly, a blood pressure cuff is placed on the forearm. Occlusion is created by inflating the cuff to at least 50 mmHg above the subject’s systolic pressure for 5 min. The pressure is then released quickly to induce hand and forearm hyperemia and subsequent brachial artery reactive vasodilation. FMD is defined as the maximal percent change in brachial artery diameter after reactive hyperemia compared with baseline. The FMD values of 60 individuals without vascular disease consisting of normal volunteers, primary headache patients, or essential tremor patients were used as reference (FMD value: 8.8 ± 2.7%, age: 57.7 ± 14.9). FMD was examined at baseline (T0) before administration of antiplatelet agents, and after 3 months of randomized antiplatelet treatment (T1).

### Specimen collection and measurement of serum l-arginine

Venous blood samples were initially collected in 3.2% sodium citrate (Becton Dickinson, USA). The serum L-arginine levels were measured by Arginine ELISA Assay Kit (Eagle Biosciences). The concentrations of analytes into the samples were estimated from the standard curve and expressed as micromole per liter.

### Statistical analysis

The statistical power of the study was set at 85%, given a mean difference of 2 and a standard deviation of 2.7 (based on our hospital laboratory results), with a type I error of 0.05 for FMD. A sample of 34 patients per group was calculated based on Dunnett’s procedure of comparing several treatments with a control (PASS 2008, USA). To allow for a 15% drop-out rate, 40 patients were randomly assigned per each group. Categorical variables were analyzed using a χ^2^ test or Fisher’s exact test. Numeric were analyzed using a Student’s *t*-test between two groups. First, comparison between the aspirin group and cilostazol group was performed to verify occurrences of adverse events and their baseline characteristics. The change of FMD values at the initial and follow-up period were compared using a paired t-test between the control and cilostazol groups. To validate the factors associated with T1 FMD values, multiple linear regression analysis was performed, including potentially significant variables in the correlation analysis (*p* < 0.200). To evaluate the effects of baseline serum L-arginine levels on FMD outcomes, correlation analysis between FMD and L-arginine levels at T0 and at T1 in both groups were compared. Statistical analyses were performed using commercially available software (SPSS, version 22.0, USA). *P*-values <0.05 were considered significant.

## Results

### Baseline clinical characteristics and adverse events

A total of 40 patients were enrolled to each group. In the aspirin group, 3 patients were excluded due to serious adverse events (SAE) (intracerebral hemorrhage, thrombocytopenia, and femur fracture), 2 patients were lost on follow up, and 1 withdrew consent. A total of 34 patients were enrolled for the final analysis. In the cilostazol group, 2 patients were excluded due to SAE (acute cholecystitis, and ischemic stroke), 1 were excluded due to adverse event (hypoglycemia), and 5 patients were lost on follow up. A total of 32 patients were included in the final analysis.

Baseline clinical factors such as age, sex, diagnosis of hypertension (HTN), diagnosis of diabetes mellitus (DM), and history of smoking did not differ between the two groups. Baseline laboratory data such as hemoglobin, glucose, lipid profiles, and serum L-arginine levels did not differ between the two groups (Table 1). When initial FMD values and mean age of aspirin and cilostazol groups were compared to reference group, there was no statistical difference (FMD values: 8.5 ± 2.6 vs. 7.9 ± 2.4 vs. 8.8 ± 2.7%, *p* = 0.257, age: 79.5 ± 11.7 vs. 57.4 ± 12.7 vs. 57.7 ± 14.9, *p* = 0.787).

**Table 1:** Baseline clinical and laboratory factors of the aspirin group and cilostazol group

	Aspirin (*n* = 34)	Cilostazol (*n* = 32)	*P*
Age	59.5 ± 11.7	57.4 ± 12.7	0.494
Male sex	20 (58.8%)	23 (71.9%)	0.266
TOAST classification			0.233
Atherosclerosis	1 (2.9%)	3 (9.4%)	
Small artery disease	28 (82.4%)	28 (87.5%)	
Unknown	3 (8.8%)	0 (0.0%)	
Transient ischemic attack	2 (5.9%)	1 (3.1%)	
HTN	28 (82.4%)	22 (68.8%)	0.197
DM	10 (29.4%)	5 (15.6%)	0.182
Cardiac problems	3 (8.8%)	2 (6.3%)	0.693
Hyperlipidemia	13 (38.2%)	14 (43.8%)	0.649
Metabolic syndrome	20 (58.8%)	14 (43.8%)	0.324
BMI	24.9 ± 3.1	24.6 ± 3.0	0.994
Smoking	16 (47.1%)	13 (40.6%)	0.599
Previous statin use	1 (2.9%)	2 (6.3%)	0.519
Laboratory data			
Hemoglobin (mg/dL)	14.0 ± 2.0	14.2 ± 1.4	0.638
Glucose (mg/dL)	147.9 ± 62.9	130.2 ± 37.8	0.173
CRP (mg/dL)	0.2 ± 0.3	0.2 ± 0.3	0.692
Fibrinogen (mg/dL)	319.4 ± 70.4	327.1 ± 78.7	0.678
Homocysteine (mg/dL)	12.0 ± 4.5	12.3 ± 6.4	0.84
L-arginine (mmol/L)	98.3 ± 42.6	95.0 ± 25.1	0.706
Lipid			
Total cholesterol (mg/dL)	192.6 ± 35.6	193.6 ± 38.6	0.913
Triglyceride (mg/dL)	157.7 ± 102.4	135.0 ± 67.4	0.296
HDL (mg/dL)	45.0 ± 12.8	44.7 ± 9.7	0.921
LDL (mg/dL)	118.5 ± 29.7	121.8 ± 33.1	0.669

*HTN* hypertension; *DM* diabetes mellitus; *BMI* body mass index; *CRP* C reactive protein; *HDL* high density lipoprotein; *LDL* low density lipoprotein

5 patients experienced SAE (intracerebral hemorrhage, thrombocytopenia, femur fracture, bell’s palsy, and infectious enterocolitis) in the aspirin group, while 2 patients experienced SAE (acute cholecystitis and ischemic stroke) in the cilostazol group (*p* = 0.235). Compared to aspirin group, the number of patients who experienced adverse events was significantly higher in the cilostazol group (62.5% vs. 87.5%, *p* = 0.01) due to higher frequency of headaches (7.5% vs. 35.0%, *p* = 0.003). The number of other adverse events did not differ (Table 2).

**Table 2:** Comparison of adverse events between the two groups

	Aspirin (*n* = 40)	Cilostazol (*n* = 40)	*P*
Serious adverse events	5	2	0.235
Acute cholecystitis	0	1	NC
Ischemic stroke recurrence	0	1	NC
Bell’s palsy	1	0	NC
Intracerebral hemorrhage	1	0	NC
Thrombocytopenia	1	0	NC
Femur fracture	1	0	NC
Infectious enterocolitis	1	0	NC
Adverse events	25	35	0.010
Headache	3	14	0.003
Anxiety/Palpitation	3	9	NC
Dizziness	2	4	NC
Heartburn /Dyspepsia	4	1	NC
Diarrhea	1	1	NC
Hand tremor	2	1	NC
Itching/skin rash	3	2	NC
Dysuria	1	0	NC
Depression	1	0	NC
Acute rhinitis	1	1	NC
Food allergy	1	0	NC
Hyperglycemia/Hypoglycemia	0	2	NC
Muscle pain	1	0	NC
Liver enzyme elevation	1	0	NC
Tinnitus	1	0	NC

### Patient management and FMD changes

Apart from randomized use of antiplatelets, other stroke managements such as blood pressure control, statins or glucose management was performed according to previously published guidelines [16]. In terms of medication, the choice of antihypertensive use did not significantly differ. While statin use was more common in the cilostazol group, all patients received lipid lowering agents. Vitamin supplementation or choice in oral hypoglycemic agents did not differ (Table 3).

**Table 3:** Treatment factors according to aspirin and cilostazol treatment groups

	Aspirin (*n* = 34)	Cilostazol (*n* = 32)	*P*
Admission MBP (mmHg)	107.6 ± 11.8	106.6 ± 15.0	0.770
3 month MBP (mmHg)	98.1 ± 11.9	95.6 ± 10.2	0.370
MBP reduction (mmHg)	9.5 ± 16.9	11.3 ± 14.5	0.659
Antihypertensives	27 (79.4%)	22 (68.8%)	0.322
ARB	27 (79.4%)	21 (65.6%)	0.209
CCB	11 (32.4%)	8 (25.0%)	0.510
BB	2 (5.9%)	0 (0.0%)	0.164
Lipid lowering agents			0.024
Statins	29 (85.3%)	32 (100.0%)	
Fibrates	5 (14.7%)	0 (0.0%)	
Vitamin Supplementation	4 (11.8%)	4 (12.5%)	0.927
Oral Hypoglycemic agents			
Metformin	8 (23.5%)	3 (9.4%)	0.123
Glimepiride	2 (5.9%)	3 (9.4%)	0.592
DPP-4 inhibitors	3 (8.8%)	1 (3.1%)	0.332

*MBP* mean blood pressure; *ARB* angiotensin receptor blockers; *CCB* calcium channel blockers; *BB* beta blockers; *DPP* dipeptidyl peptidase

**Fig. 1:**
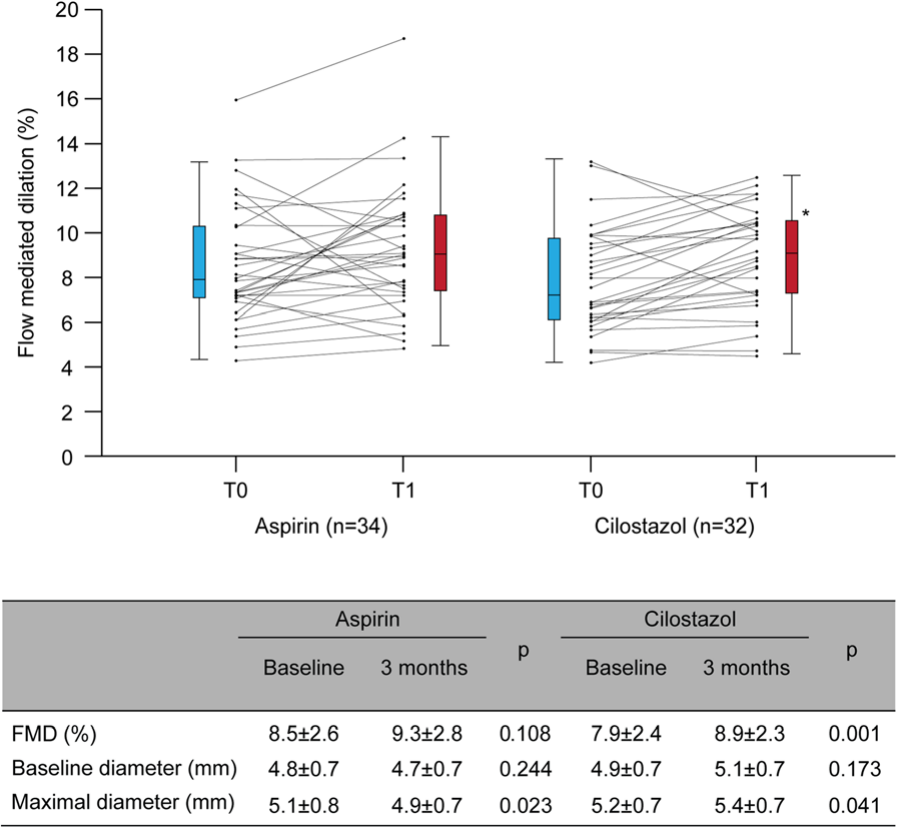
The FMD parameters on enrollment and at 3 months. A significant increase of FMD values in the cilostazol group is seen, while not in the aspirin group

Despite no difference in the baseline FMD between the groups (*p* = 0.363), there was a significant increase of FMD values in the cilostazol group (7.9 ± 2.4 to 8.9 ± 2.3%, *p* = 0.001) while not in the aspirin group (8.5 ± 2.6 to 9.3 ± 2.8%, *p* = 0.108). Increase in maximal brachial artery diameter was also seen in the cilostazol group (5.2 ± 0.7 to 5.4 ± 0.7, *p* = 0.041) (Fig. 1).

### Factors associated with FMD at T1 in cilostazol group

In the multiple linear regression analysis for evaluation of factors associated with increase in FMD values at T1, factors that were potentially associated with FMD levels in the correlation analysis (*p* < 0.200) were included (Table 4). In the multivariate analysis, baseline L–arginine levels were inversely correlated with FMD at T1 (ß = −0.050, SE: 0.012, *p* < 0.001), as also seen in baseline C-reactive protein levels (ß = −3.267, SE = 0.984, *p* = 0.001) (Table 4).

**Table 4:** Factors that are correlated with 3 month FMD values of the cilostazol group in multiple linear regression analysis

	Univariate analysis		Multivariate analysis (r^2^ = 0.695)			
Factors	r	p	ß	SE	95% CI	p
Age	–.286	0.113	–0.036	0.024	(–0.086, 0.013)	0.145
Initial mean BP	.003	0.988				
3 month mean BP	–.007	0.968				
Hemoglobin	.003	0.988				
Glucose	.122	0.507				
Total cholesterol	.249	0.170	0.009	0.008	(–0.008, 0.025)	0.290
Triglyceride	.162	0.376				
HDL	.017	0.927				
LDL	.219	0.228				
ESR	–.004	0.982				
CRP	–.352	0.048	–3.267	0.984	(−5.285, –1.248)	0.003
L-arginine	–.508	0.003	–0.050	0.012	(−0.075, –0.024)	<0.001

*FMD* flow mediated dilation; *BP* blood pressure; *HDL* high density lipoprotein; *LDL* low density lipoprotein; *ESR* erythrocyte sedimentation rate; *CRP* c-reactive protein

### Serum L-arginine levels and FMD

**Fig. 2:**
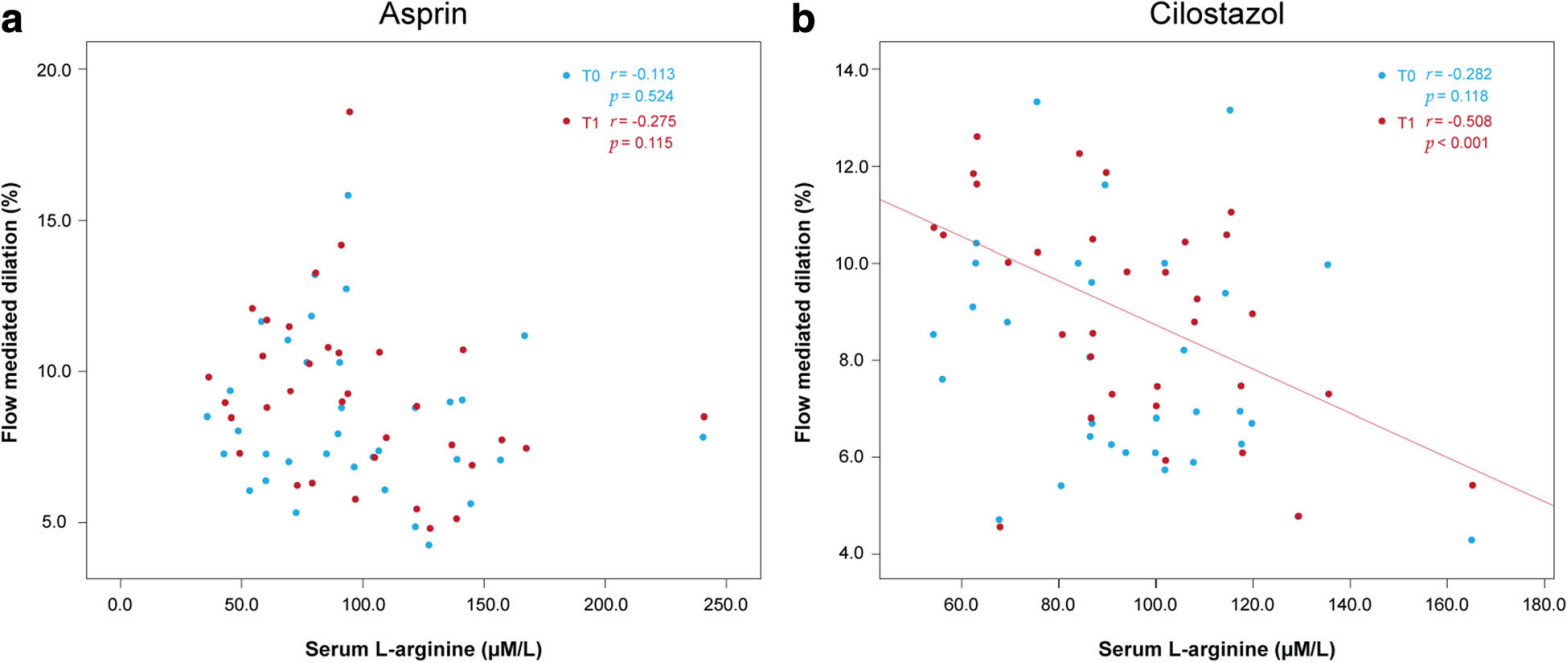
Correlation between initial serum L-arginine levels and FMD at enrollment and at 3 months. Blue dots denote FMD at enrollment (T0), and red dots denote FMD at 3 months (T1). **a** In the aspirin group FMD values and L-arginine levels do not show correlation at T0 and at T1. **b** In the cilostazol group, T0 FMD values and L-arginine levels do not show correlation, but an inverse correlation between the two values is revealed at T1

Figure 2 shows the correlation analysis between serum L-arginine levels and FMD at initial and 3 month. At T0, both cilostazol (*r* = 0.231, *p* = 0.204) and aspirin (*r* = 0.26, *p* = 0.883) groups do not show correlation between serum L-arginine levels and FMD. At T1, cilostazol group shows restored correlation between serum L-arginine levels and FMD (*r* = 0.467, *p* = 0.007), while such is not shown in the aspirin group (*r* = 0.174, *p* = 0.324).

## Discussion

To the best of our knowledge, this is the first study to investigate the effect of cilostazol on endothelial function by FMD in patients with acute cerebral ischemia. Our study results show that administration of cilostazol had a beneficial effect on endothelial function in acute cerebral ischemia patients. Furthermore, cilostazol treated patients showed significant relationship between baseline serum L-arginine levels and FMD, while such findings are not seen in the aspirin group. Our study results emphasize the use of cilostazol in acute ischemic stroke [17], for cilostazol induced improvements in endothelial function may be an underlying surrogate marker for its role in prevention of atherogenesis [6] and blocking small-vessel pathology [18].

Previous studies on FMD and cilostazol have shown conflicting results. Results of studies involving smokers have shown improvement in endothelial function, and younger smokers are more likely to respond to treatment [19–21]. Preclinical studies supporting such findings have shown that cilostazol increases NO synthesis in interleukin-1 beta-stimulated smooth muscle cells, at least partially through a cAMP-dependent pathway [22, 21]. Furthermore, cilostazol increased production of NO and concentration dependent vasodilation in porcine thoracic aorta. This finding was more obvious with intact epithelium compared to denuded aorta, suggesting that cilostazol-induced vasodilation was dependent on endothelial release of NO [23].

However, an FMD study involving Raynaud syndrome patients [24] and a recent study involving coronary artery disease patients [25] both showed no improvement of FMD with oral cilostazol use for 6 weeks and 6–9 months respectively, while significant improvement in brachial artery diameter was seen in both studies. The negative results of the two recent disease oriented studies represent the multifactorial causes of endothelial dysfunction encountered in clinical settings. Patients with overt coronary artery disease or peripheral arterial obstructive disease have multiple vascular risk factors such as dyslipidemia [26], hypertension [27], or diabetes [28] that lead to impaired NO bioavailability by multiple mechanisms such as endothelial nitric oxide synthase (eNOS) uncoupling, increased NO breakdown by reactive oxygen species, or increase in asymmetric dimethylarginine (ADMA) [29]. A single agent such as cilostazol may not be sufficient for improvements in endothelial function in such complex situations, and furthermore, the effect of cilostazol may have been masked by other medications known to have effect on endothelial function. In this regard, the positive results seen in our study may have been due to the somewhat homogenous inclusion criteria of patients that had not used antithrombotic agents previously.

A subsequent result of our study is the inverse correlation seen between serum L-arginine levels and FMD in the cilostazol group after 3 months. One might presume that higher levels of L-arginine, a substrate for NO, may be associated with higher FMD values. However, endothelial function is known to be more dependent on ADMA levels, a competitive inhibitor of NO synthase; namely the L-arginine paradox. Furthermore, because L-arginine-dependent metabolic pathways are critical determinants of several pathophysiological conditions, its levels are generally tightly regulated [30]. In hypertensive patients [31] and end-stage renal disease patients [32], endothelium dependent vasodilation showed inverse relations with plasma L-arginine levels along with ADMA levels [31]. In the case of hypercholesterolemia, serum L-arginine levels did not differ much in contrast to ADMA levels showing inverse correlation with endothelial function [33]. Overall, plasma L-arginine levels seem to show inverse relation with endothelial function. Such inverse relation was seen only in the 3 month cilostazol group, while this relation was altered in the acute stroke phase of both population, and 3 month aspirin group. Thus, only the 3 month FMD values of cilostazol group are in line with the L-arginine paradox. While our study results itself lack further explanation, we presume that accumulation of multiple risk factors and acute ischemia systemically affects endothelial function, resulting in alteration of normal endothelial physiology. Cilostazol seems to induces improvements in suppressed eNOS bioactivity by phosphorylation of activation sites [34], restoring the physiologic relation between plasma L-arginine levels and FMD, while such finding is not seen in the aspirin group.

Our study enlightens another clinically important issue of pharmacotherapy. The use of cilostazol and L-arginine co-supplementation. Although physiological range of L-arginine are in a range that enables full activity of its enzyme [30], there is still evidence that oral supplementation doses improve endothelial function in certain patient groups [35, 36]. By combined use of L-arginine supplementation and cilostazol, further improvements in endothelial function may be achieved, for L-arginine acts as a substrate for NO synthesis, while cilostazol activates eNOS via phosphorylation of its activation sites. Further clinical studies may be needed to address this issue.

The current study has some limitations. First, plasma L-arginine levels were measured only on admission due to limitations in study design. Accordingly, the serial changes in its values and its association with FMD values could not be evaluated. Also, serum ADMA levels were not measured in our study. However, previous studies have revealed that plasma L-arginine levels are known to correlate with plasma ADMA levels [31], and previous reports that L-arginine supplementation successfully improved the FMD in special populations [33] support our study results. We hope to address these limitations in future studies using cilostazol and L-arginine co-supplementation. Another limitation is the small number of patients included, that led to intergroup difference. While insignificant, the percentage of patients with comorbidity of DM and HTN tends to be higher in the aspirin group, which may have biased the results in favor of cilostazol group. In terms of lipid lowering therapy, statin use was higher in the cilostazol group, while all patients received lipid lowering agents. This may have influenced our results, for statin therapy can be associated with improvements in endothelial function [37]. In interpretation of the FMD values, while insignificant, the initial FMD values tended to be higher in the aspirin group, and this baseline difference may have affected the FMD changes. Considering the reference FMD values of our lab, there may be chance that elevations in FMD values of both groups reach a plateau at some point, and the lower baseline FMD values in cilostazol group may have biased the results. However, to our knowledge, no previous study addressing improvements of FMD have considered the issue of FMD reaching plateau levels, and such issues need to be clarified in future studies.

## Conclusions

In acute cerebral ischemia patients, we compared the changes of FMD after 3 month double blinded randomized treatment with aspirin and cilostazol. Cilostazol improved endothelial function in acute cerebral ischemia patients, but aspirin did not. It also restored an inverse correlation between 3-month FMD and baseline L-arginine levels. The action of cilostazol in its roll of improving endothelial function seems to be due to activation of eNOS, as previous studies have shown. Our study emphasizes the role of cilostazol in the acute phase of cerebral ischemia, as it holds its roll in stabilization of dysfunctional endothelium, and remodeling of vessel wall structure. L-arginine and cilostazol co-administration may be considered for further improvement in endothelial function.

## Additional file

Additional file 1: Figure S1. A flowchart for the study population. (TIFF 178 kb)

## Abbreviations

ADMA: asymmetric dimethylarginine
cAMP: cyclic adenosine monophosphate
DM: diabetes mellitus
eNOS: endothelial nitric oxide synthase
FMD: flow mediated dilation
HTN: hypertension
NO: nitric oxide
PDE: phosphodiesterase
SAE: serious adverse events
TIA: transient ischemic attack
