# Supplementary figures and images for: Cilostazol improves endothelial function in acute cerebral ischemia patients: a double-blind placebo controlled trial with flow-mediated dilation technique
Source: BMC Neurol. 2017 Aug 29;17:169. doi: 10.1186/s12883-017-0950-y (PMC5576326; doi:10.1186/s12883-017-0950-y)

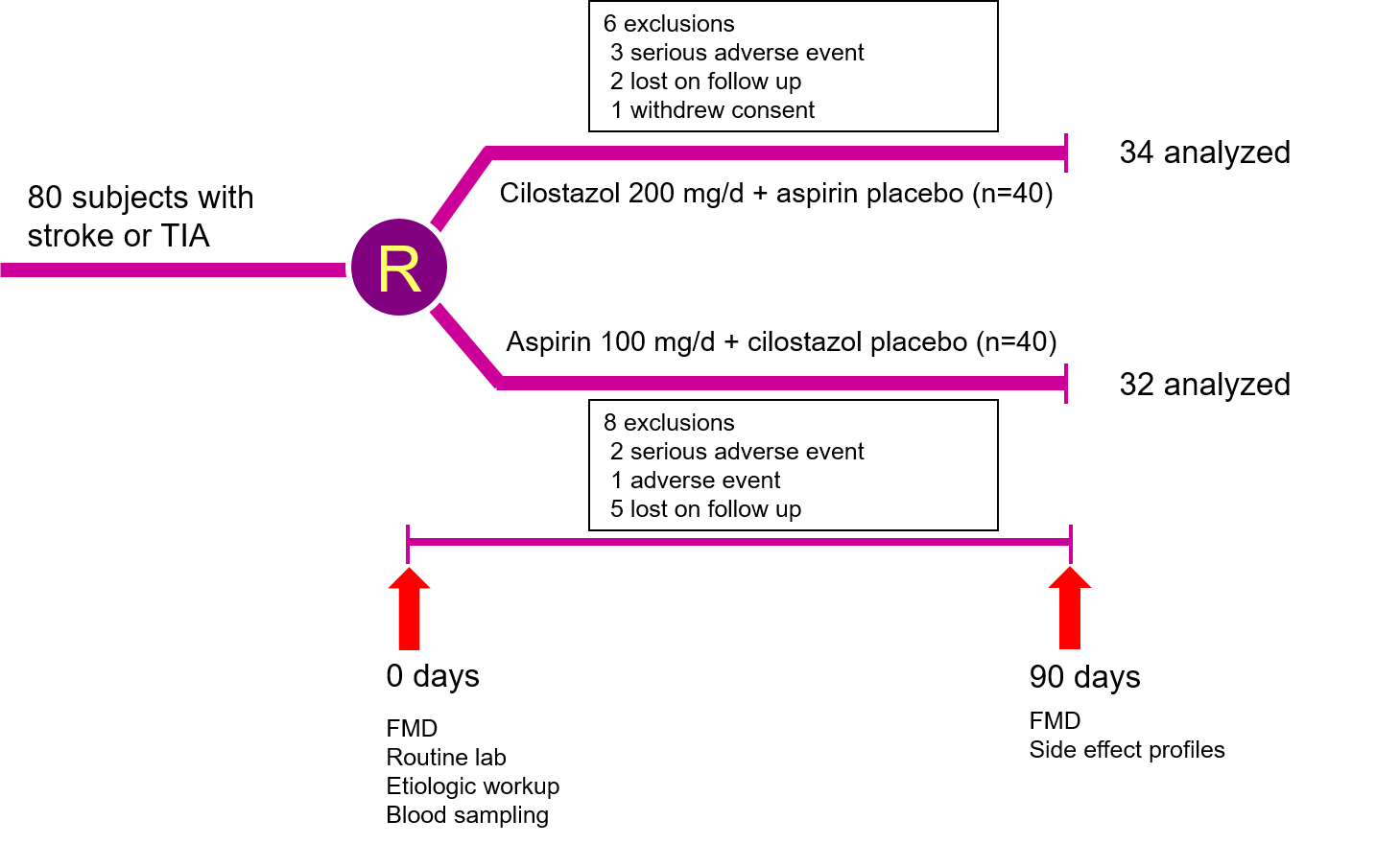

Supplement: Additional file 1: Figure S1. — A flowchart for the study population. (TIFF 178 kb) [file 12883_2017_950_MOESM1_ESM.tif]
